# Anergy into T regulatory cells: an integration of metabolic cues and epigenetic changes at the Foxp3 conserved non-coding sequence 2

**DOI:** 10.12688/f1000research.16551.1

**Published:** 2018-12-17

**Authors:** Milagros Silva Morales, Daniel Mueller

**Affiliations:** 1 Division of Rheumatic and Autoimmune Diseases, Center for Immunology, and the University of Minnesota Medical School, Minneapolis, USA

**Keywords:** Anergy, Treg differentiation, CNS2 methylation, Peripheral tolerance, Foxp3

## Abstract

Peripheral immune self-tolerance relies on protective mechanisms to control autoreactive T cells that escape deletion in the thymus. Suppression of autoreactive lymphocytes is necessary to avoid autoimmunity and immune cell–mediated damage of healthy tissues. An intriguing relationship has emerged between two mechanisms of peripheral tolerance—induction of anergy and Foxp3 ^+^ regulatory T (Treg) cells—and is not yet well understood. A subpopulation of autoreactive anergic CD4 T cells is a precursor of Treg cells. We now hypothesize that phenotypic and mechanistic features of Treg cells can provide insights to understand the mechanisms behind anergy-derived Treg cell differentiation. In this short review, we will highlight several inherent similarities between the anergic state in conventional CD4 T cells as compared with fully differentiated natural Foxp3 ^+^ Treg cells and then propose a model whereby modulations in metabolic programming lead to changes in DNA methylation at the Foxp3 locus to allow *Foxp3* expression following the reversal of anergy.

## Introduction

Foxp3 ^+^ regulatory T (Treg) cells and the induction of anergy in conventional CD4 T cells each represent peripheral tolerance mechanisms designed to control autoreactive CD4 T cells that escape negative selection in the thymus ^1^. Naturally occurring Treg cells are regularly generated in the thymus when a thymocyte encounters a high-affinity self-peptide/MHC II ligand and gains the expression of CD25 ( *Il2ra*) and Foxp3 ( *Foxp3*) ^2, 3^. Once differentiated in the thymus, natural Foxp3 ^+^ Treg cells move to the periphery where their purpose is to maintain conventional T-cell homeostasis through a process called suppression ^3^. In contrast, anergy is established in the periphery when a conventional CD4 T cell recognizes a self-peptide/MHC II complex in the absence of infection or adjuvant ^4^. Anergy in the normal polyclonal CD4 T-cell repertoire leads to a state of functional unresponsiveness characterized by a block in autocrine growth factor production—for example, interleukin-2 (IL-2)—that prevents dangerous autoimmune responses ^5^. An interesting and close relationship between these two tolerance mechanisms has recently emerged, and it has been demonstrated that naturally occurring anergic polyclonal CD4 T cells contain a subpopulation of Treg progenitors that can differentiate into the Foxp3 ^+^ Treg lineage ^5^. However, the physiological and biochemical mechanisms responsible for this generation of anergy-derived Foxp3 ^+^ Treg cells remain uncertain.

## The differentiation of Foxp3 ^+^ Treg cells

*Foxp3* gene expression defines the Treg lineage in mice and is essential to its counter-regulatory activities ^6^. Both mice and humans lacking expression of a normal *Foxp3* allele demonstrate spontaneous and potentially lethal autoimmune disease ^7– 9^. Foxp3 acts mainly as a transcriptional repressor during periods of inflammation, and a large fraction of its inhibited target genes are important for T-cell receptor (TCR) signaling, transcriptional activation, and chromatin remodeling ^10, 11^. Foxp3 ^+^ Treg cells cannot initiate autocrine growth factor production and proliferation yet demonstrate an ability to respond to IL-2 and other pro-inflammatory stimuli in a paracrine fashion to suppress the proliferation of dangerous conventional CD4 T cells ^12, 13^.

Floess *et al*. ^14^ were the first to demonstrate that stable expression of the *Foxp3* gene in Treg cells is associated with alterations in DNA methylation. A Treg-specific demethylated region (TSDR) enhancer element upstream of the *Foxp3* promoter that contains a CpG island is uniquely unmethylated in natural Foxp3 ^+^ Treg cells. Soon thereafter, Kim and Leonard ^15^ identified two additional *Foxp3* CpG island–containing conserved non-coding sequences (CNS1 and CNS3) that were also fully unmethylated only in Treg cells. Interestingly, the stimulation of conventional Foxp3 ^–^ CD4 T cells with the combination of CD3 and CD28 monoclonal antibodies plus IL-2 in the presence of either transforming growth factor-beta (TGF-β) or the DNA methyltransferase (DNMT) inhibitor 5-azacytidine was found to be sufficient to induce partial demethylation of these TSDR, CNS1, and CNS3 regions in association with new expression of Foxp3 ^15, 16^.

Complete demethylation of one other CpG island within the intronic *Foxp3* CNS2 cis-acting element is now also understood to be key to maintaining the expression of the lineage-defining Foxp3 transcription factor in CD4 T cells ^17^. Ohkura *et al*. ^18^ showed that the establishment of a stable Foxp3 ^+^ Treg cell lineage requires both strong and continuous TCR stimulation during development. TCR engagement by self-peptide/MHC II triggers the initial transcription of *Foxp3*, whereas prolonged TCR signaling allows for the development of a natural Treg demethylation (nTreg-Me) signature that is associated with stable *Foxp3* expression. This nTreg-Me signature is characterized as complete or near complete demethylation of CpG islands in *Il2ra*, *Ctla4*, *Ikzf4*, and *Tnfrsf18* as well as the *Foxp3* CSN2 itself. Whereas *de novo* Foxp3 ^+^ Treg cell differentiation, survival, activation, and effector function depend on continuous TCR engagement and downstream signaling, the TCR itself ultimately becomes irrelevant either for the maintenance of *Foxp3* gene expression or for the demethylation signature seen in stable natural Foxp3 ^+^ Treg cells ^19^. Thus, demethylation of the *Foxp3* CNS2 appears to be uniquely important to the stable expression of Foxp3 and the maintenance of Treg cell suppressor function.

## The intersection between cellular metabolism and *Foxp3* CNS2 methylation/demethylation by DNA methyltransferases and ten-eleven translocation proteins

Data suggest that a balance between the activities of the DNMTs and the ten-eleven translocation (TET) proteins directly controls the state of CNS2 CpG methylation and the stability of *Foxp3* gene expression. During the S phase of the cell cycle, DNMT1 can be expected to recognize hemi-methylated CNS2 CpG sequences when a replication fork enters the *Foxp3* locus to catalyze the “maintenance methylation” of the newly replicated daughter DNA strand ^20^. Once chromosomal replication ceases, a complex of DNMT1 and DNMT3b has the opportunity to bind 5-methylcytosines within the *Foxp3* locus to promote the “ *de novo* methylation” of any nearby unmethylated CpG groups ^20, 21^. Therefore, DNMT activity represents a significant potential barrier to CNS2 CpG demethylation and stable Foxp3 ^+^ expression. Nonetheless, during Treg cell differentiation, TET proteins compete with DNMT1 for binding to 5-methylcytosine and catalyze the oxidation of 5-methylcytosine to 5-hydroxymethylcytosine, ultimately leading to the complete demethylation of *Foxp3* CpG islands in daughter cells during the course of cell cycle progression ^22, 23^. Perhaps consistent with such antagonism between DNMT1 and TET in Treg cells, knockdown of DNMT1 activity induces the expression of *Foxp3* in conventional CD4 T cells whereas loss of TET protein activity leads to unstable *Foxp3* expression ^15, 22– 25^.

Both DNMT1 and TET enzymatic activities are highly sensitive to the metabolic state of T cells. Unlike T effector (Teff) cells that rely heavily on aerobic glycolysis for energy generation, stable Foxp3 ^+^ Treg cells generate little lactate in the presence of glucose and instead make use of lipid and glucose oxidative phosphorylation (OXPHOS) and mitochondrial electron transport for ATP synthesis ^26, 27^. Initial *Foxp3* expression and Treg differentiation appear independent of phosphatidylinositol 3-kinase, Akt, and mechanistic target of rapamycin (mTOR) signaling, and mature natural Treg cells continue to demonstrate only low mTOR activity in the resting state ^13, 28^. Expression of neuropilin 1 (Nrp1) and Foxp3 on Treg cells reinforces this low mTOR activity, thus restricting aerobic glycolysis during periods of immune homeostasis ^12, 29^. Nevertheless, the activation and cell cycle progression of short-lived “effector” Treg cells require an increase in mTOR activity and the induction of aerobic glycolysis ^12, 30^. mTOR mediates the upregulation of the glucose transporter Glut1 and other nutrient transporter systems and orchestrates the shift in cellular metabolism away from OXPHOS toward aerobic glycolysis. In addition, mTOR promotes the biogenesis of mitochondria through its control of mRNA translation ^31, 32^. Both co-stimulatory receptors such as CD28 and γc-chain cytokines such as IL-2 and IL-7 trigger signaling to mTOR and drive this change in metabolic program ^33– 35^.

DNMT1-dependent DNA methylation depends on high levels of the methyl group donor S-adenosyl methionine (SAM-e), whose cellular concentration in turn is controlled by the activity of the nutrient-sensitive (that is, vitamin B _12_, folic acid, methionine, serine, and glutamine) one-carbon and SAM-e metabolic pathways ^36^. Following entry into the cell cycle, T cells generally shift from a dependence on oxidative phosphorylation (OXPHOS) to an anabolic metabolic state that relies on both the upregulation of aerobic glycolysis and new mitochondrial biogenesis ^37^. In particular, mitochondrial enzymes and co-factors necessary for one-carbon metabolism are upregulated prior to the first G _1_-to-S phase transition to facilitate the conversion of homocysteine to methionine and ultimately SAM-e ^37^. Activity of TET, in contrast to DNMT1, depends on the citric acid cycle intermediate alpha-ketoglutarate (α-KG) to act as a co-factor in 5-methylcytosine oxidation ^38^. The induction of enzyme activities associated with glutaminolysis during the initiation of cell cycle progression similarly leads to an increase in the generation of α-KG ^39, 40^. Thus, proliferating T cells are subject to dramatic increases in enzyme activities important to both DNA methylation and demethylation and this contributes to their differentiation plasticity during cell cycle progression.

## Anergic CD4 T cells are Treg progenitors

One long-standing question in the investigation of anergy as a peripheral immune tolerance mechanism has been its purpose. Why should the immune system actively promote the survival of potentially dangerous self-reactive T cells when mechanisms exist to delete such cells from the repertoire? One attractive hypothesis is that anergy reversal can at times be protective—either to facilitate aggressive immunity against particular tissue-specific self-antigens during intracellular infection or cancer or to augment antigen-specific suppression in the face of immunopathology. In support of the latter hypothesis, anergic Foxp3 ^–^ TCR-transgenic CD4 T cells specific for influenza hemagglutinin (HA) that were recovered from HA-expressing double-transgenic hosts demonstrated an ability to produce IL-10 and suppress normal CD4 T-cell reactivity against an adenovirus vector–delivered HA immunization ^41, 42^. Similarly, Foxp3 ^–^ male antigen *Dby*-specific TCR-transgenic CD4 T cells induced into anergy following adoptive transfer into male mice developed the capacity to suppress naïve T-cell responses to *Dby* both *in vitro* and *in vivo* ^43^. Finally, chicken ovalbumin (OVA)-specific TCR-transgenic CD4 T cells made anergic following *in vitro* stimulation with OVA-loaded immature bone marrow–derived dendritic cells also acquired a Tr1-like suppressive phenotype after repeated stimulation in association with the upregulation of *Egr2*, CTLA-4, IL-10, and CD25 but not Foxp3 ^44^.

Our recent discovery of a repertoire of naturally occurring anergic CD4 Treg cell progenitors in healthy mice has provided the opportunity to further explore this relationship between anergy induction and immunoregulation ^5, 45^. Anergic polyclonal Foxp3 ^–^ FR4 ^+^ CD73 ^+^ Nrp1 ^+^ CD4 T cells from *Foxp3 ^DTR-GFP^* mice were shown to lose their anergy markers and undergo cell cycle progression when transferred to *Tcra* ^–/–^ hosts lacking their own Foxp3 ^+^ Treg compartment. However, recipient mice seldom exhibited evidence of autoimmune disease because a subset of these donor anergic T cells eventually differentiated into the Foxp3 ^+^ Treg cell lineage accounting for as many as 20% of the descendent T cells. This was in contrast to mice treated with diphtheria toxin during the anergy reversal to inhibit the accumulation of anergy-derived Foxp3 ^+^ Treg cells, as these mice uniformly developed colitis associated with weight loss and generated autoantibodies that recognized gut, heart, liver, lung, salivary gland, kidney, and pancreas antigens in an organ-specific fashion. The formal proof that such polyclonal anergy-derived Foxp3 ^+^ Treg cells could be protective was obtained by using *in vivo* models of inflammatory bowel disease and autoimmune arthritis in which the adoptive transfer of anergy-derived Foxp3 ^+^ Treg cells suppressed disease development ^5^. Thus, anergy reversal and cell cycle progression in Treg-deficient *Tcra ^–/–^* hosts were associated with the differentiation of anergic CD4 T cells into Foxp3 ^+^ Treg cells that could protect the recipients from immunopathology.

Unlike the Tr1-like Foxp3 ^–^ Treg cells previously generated from TCR-transgenic anergic T cells, polyclonal anergy-derived Treg cells were shown to stably express both Foxp3 and Nrp1 ^5^. Importantly, anergy-derived Nrp1 ^+^ Foxp3 ^+^ Treg cells also demonstrated a fully demethylated nTreg-Me gene signature (including CpG islands in *Ctla4*, *Tnfrsf18*, *Ikzf4*, and the *Foxp3* CNS2 locus) similar to thymic Foxp3 ^+^ Treg cells. In contrast, the dangerous anergy-derived Teff cells that failed to upregulate *Foxp3* expression had a completely methylated *Foxp3* CNS2 region after anergy reversal. Experiments established that some anergic Foxp3 ^–^ FR4 ^+^ CD73 ^+^ Nrp1 ^+^ polyclonal CD4 T cells in healthy *Foxp3 ^DTR-GFP^* knockin mice had undergone incomplete demethylation of CpG islands in the nTreg-Me signature genes. In particular, about half of the *Ctla4* exon 2 sequenced DNA alleles were found to be demethylated on at least three of the five CpG nucleotides, a pattern not observed in any other subset within the conventional CD4 polyclonal T-cell repertoire. Therefore, we now hypothesize that partial demethylation of the *Foxp3* CNS2 region is a key feature of anergic Treg progenitor cells.

## A two-step model for anergy-derived Foxp3 ^+^ Treg differentiation

At this time, the molecular mechanisms that dictate the generation of anergy-derived Foxp3 ^+^ Treg cells versus acquisition of a dangerous Teff cell phenotype remain unknown. However, natural Foxp3 ^+^ Treg cells may offer important clues as they can demonstrate similar lineage plasticity during periods of lymphopenia-induced proliferation or target tissue inflammation (for example, excess IL-6 and IL-23) where they risk the loss of both *Foxp3* expression and suppressor activity (the so-called exFoxp3 cells) ^46, 47^. CD25 ^lo^ Helios ^–^ Nrp1 ^–^ Foxp3 ^+^ Treg cells with their incomplete *Foxp3* CNS2 CpG demethylation in particular are prone to loss of *Foxp3* expression ^29, 46– 49^. We note that, similar to anergy reversal in *Tcra ^–/–^* hosts, each of these pathophysiological settings that favor Treg lineage instability and the generation of exFoxp3 cells is marked by a period of TCR-mediated cell cycle progression. Therefore, DNMT1 activity and *Foxp3* CNS2 maintenance methylation may destabilize *Foxp3* expression in the setting of T-cell lymphopenia. Nevertheless, we understand that exFoxp3 cells can regain their capacity to express the *Foxp3* gene and suppress CD4 T-cell responses following proliferation in the presence of IL-2 ^46, 50^. This predicts a role for CD25-triggered activation of mTOR-dependent glutaminolysis and aerobic glycolysis and the resultant upregulation of α-KG/TET-mediated antagonism of DNMT1 function during chromosomal replication. This shift away from OXPHOS metabolism may also reduce the availability of oxidized nicotinamide adenine dinucleotide (NAD ^+^) ^51^ and prevent the NAD-dependent deacetylase sirtuin 1 (Sirt1) from marking Foxp3 molecules for proteasomal degradation ^52, 53^. These observations, therefore, may serve as a useful paradigm for the generation of anergy-derived Foxp3 ^+^ Treg cells, as described in the model below.

## Model step 1

**Figure 1.**
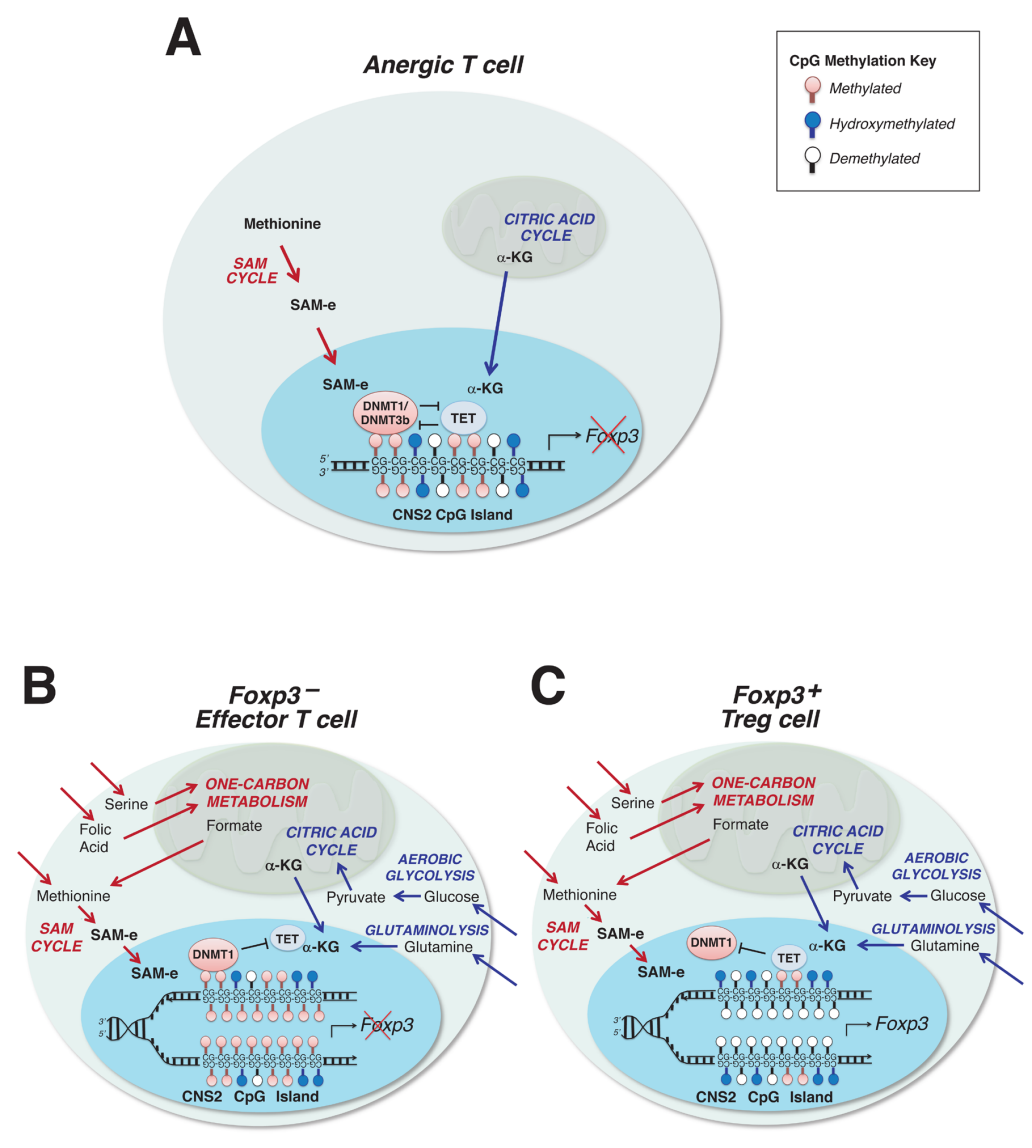
Anergy induction and anergy-derived Foxp3 ^+^ Treg cell differentiation in a two-step model. ( **A**) Anergy induction creates Treg cell progenitors with a partially demethylated nTreg-Me signature as a consequence of balanced DNMT1/DNMT3b methyltransferase and TET dioxygenase activities. ( **B, C**) Anergy reversal is associated with changes in metabolism that control DNA methylation. Dominant DNMT1 function during chromosomal replication ( **B**) generates daughter cells with fully methylated *Foxp3* CNS2 CpG islands that differentiate into Foxp3 ^–^ effector T cells, whereas dominant TET activity promotes fully demethylated daughter cells ( **C**) that differentiate into Foxp3 ^+^ Treg cells. α-KG, alpha-ketoglutarate; CNS, conserved non-coding sequence; DNMT, DNA methyltransferase; SAM-e, S-adenosyl methionine; TET, ten-eleven translocation; Treg, regulatory T.

**Figure 2.**
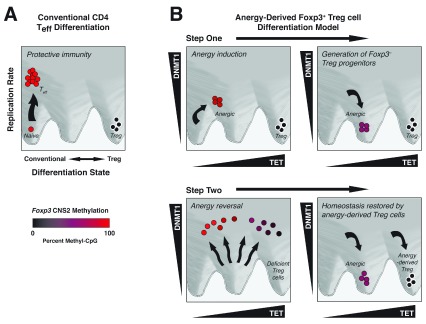
Varying DNMT1 and TET protein activities during cell cycle progression control the differentiation state of CD4 T cells. ( **A**) Conventional CD4 T effector (Teff) cell differentiation occurs in highly proliferative cells with unopposed DNMT1 activity maintaining a fully methylated *Foxp3* CNS2 region. ( **B**) Anergy-derived Foxp3 ^+^ Treg cell differentiation is a two-step process. At the end of step 1, anergic T cells undergo partial demethylation at the CNS2 locus as a result of TET dioxygenase activity. Those anergic cells that accumulate the highest number of CNS2 CpG demethylation events become resistant to DNMT1 activity during anergy reversal. In step 2, such anergic Treg cell progenitors fully demethylate their natural Treg demethylation (nTreg-Me) signature genes during chromosomal replication to promote their differentiation to the Foxp3 ^+^ Treg cell lineage. CNS, conserved non-coding sequence; DNMT, DNA methyltransferase; TET, ten-eleven translocation; Treg, regulatory T.

We hypothesize that, in the first step of this model, anergic Foxp3 ^–^ FR4 ^+^ CD73 ^+^ Nrp1 ^+^ CD4 T cells are prone to steady-state partial demethylation of nTreg-Me signature genes ( Figure 1A). Despite the opportunity for DNMT1-dependent *Foxp3* CNS2 CpG maintenance methylation early during the course of anergy induction, sustained α-KG/TET dioxygenase activity likely opposes the actions of DNMT1 and DNMT3b once anergy develops and proliferation ceases ( Figure 2). As described above, mTOR activity is highly restricted in anergic T cells by the absence of IL-2, low CD28 co-stimulatory signaling, and the presence of Nrp1 during the induction of anergy. This results in a cellular metabolism that is biased toward a reliance on OXPHOS for ATP and NAD ^+^ generation more so than aerobic glycolysis ^54, 55^. Restricted mTOR-dependent anabolic signaling pathways, including the nutrient transport systems for glucose, lipids, vitamins, and essential amino acids (for example, methionine), may be expected to reduce one-carbon metabolism and the availability of SAM-e, thus reinforcing mTOR inactivation via the SAMTOR SAM-e nutrient sensor ^56^. This low SAM-e abundance may also interfere with DNMT1/DNMT3b-dependent *de novo* methylation at the CNS2 locus of anergic CD4 T cells. DNMT1 recognition of CNS2 5-methylcytosine nucleotides may also be adversely affected by the binding of TET proteins at the locus. Finally, the sustained level of OXPHOS mitochondrial metabolism observed in anergic T cells may be sufficient to ensure adequate levels of α-KG to support TET protein oxidation and allow for the partial demethylation of CpG nucleotides within the CNS2 locus.

## Model step 2

Although this first step in the model generates Treg progenitor cells from conventional anergic CD4 T cells, our data suggest that this single step is insufficient to induce differentiation to the Foxp3 ^+^ Treg lineage. Abundant NAD ^+^ and Sirt1 deacetylase activity present in anergic T cells likely prevent any accumulation of Foxp3 protein despite partial CNS2 demethylation ^57, 58^. Anergic Treg progenitors must additionally undergo a period of anergy reversal to enter a more plastic state that facilitates the differentiation of Foxp3 ^+^ Treg cells ( Figure 2). Such a two-step system ensures that potentially autoreactive anergic T cells are called out to undergo a clonal expansion only when relevant self-antigen–specific Foxp3 ^+^ Treg suppression is insufficient, thus preserving the balance between self-tolerance, immunity, and immunodeficiency.

In step 2 of this model, anergy reversal occurs after a period of cell cycle progression in response to TCR signaling plus a new mTOR-dependent shift in cellular metabolism away from OXPHOS and toward aerobic glycolysis. Self-peptide/MHC II complex recognition is a consistent feature of the anergic state and continues to be essential during anergy reversal, as the loss of TCR signaling simply leads to the disappearance of cells ^5^. mTOR activation is best achieved in anergic T cells by TCR engagement in the setting of an acute reduction in the host’s natural Foxp3 ^+^ Treg cell repertoire. Either *Tcra ^–/–^* hosts deficient for all Treg cells or *Foxp3 ^DTR-GFP^* mice treated with diphtheria toxin to acutely deplete *Foxp3*-expressing Treg cells have been found to optimally support anergy reversal and anergy-derived Treg generation ^5^ (unpublished observations, M. Silva Morales). We would suggest that anergy reversal is triggered by the pro-inflammatory milieu that accompanies a deficiency of functional Foxp3 ^+^ Treg cells and by a lack of Treg competition for relevant self-peptide/MHC II complexes, growth factors (for example, IL-2 and IL-7), nutrients, and co-stimulatory signals. mTOR becomes activated in this setting, leading to cell cycle progression (as evidenced by increasing Ki-67 expression and clonal expansion), chromosomal replication, and accompanying anergy reversal, including the loss of expression of FR4 and CD73 and the restoration of effector cytokine production in some daughter T cells ^5^.

One expected effect of this mTOR-dependent cell cycle progression is an increase in DNMT1 maintenance methylation activity. Increased serine, methionine, and folic acid uptake during anergy reversal would ensure optimal generation of SAM-e and high DNMT1 methyltransferase function. As a consequence, in some cells, methylated CpG nucleotides that persist within the *Foxp3* CNS2 locus in anergic CD4 T cells will be recognized by DNMT1 to allow for the propagation of repressive fully methylated CpG epigenetic marks during DNA replication. Thus, we hypothesize that high DNMT1 and SAM-e levels, chromosomal replication, and *Foxp3* CNS2 remethylation promote the differentiation of dangerous conventional CD4 Teff cells following the reversal of anergy. This is particularly true when an anergic T cell has accumulated only a modest number of hydroxymethylated and demethylated CpG nucleotides at the CNS2 locus ( Figure 1B and Figure 2). Nevertheless, in this model, mTOR-dependent aerobic glycolysis, glutamine transport, and glutaminolysis are also upregulated to ensure optimal α-KG levels and TET dioxygenase activity. As a result, TET proteins can act to inhibit the recognition of hemi-methylated CNS2 DNA by DNMT1 during replication and ultimately allow for the generation of fully demethylated daughter cells that express Foxp3 at the completion of cell cycle progression. Accordingly, this model suggests that TET-dependent hydroxymethylation and demethylation events that had previously accumulated at the *Foxp3* CNS2 region in step 1 will favor the complete demethylation of the locus at the end of cell cycle progression ( Figure 1C). Once homeostasis is restored in anergy-derived Foxp3 ^+^ Treg cells and OXPHOS metabolism is re-established, remethylation of the locus by DNMT1 can once again be inhibited by reduced SAM-e levels as well as increased competition for CNS2 methylcytosine binding by active α-KG/TET dioxygenase complexes.

## Conclusions

Although the mechanisms responsible for conversion of anergic cells into Treg cells remain unclear, the phenotypic and biochemical similarities between anergic T cells and natural Foxp3 ^+^ Treg cells provide important clues. Two shared traits, in particular, may be important to the differentiation of anergy-derived Treg cells: (a) OXPHOS metabolism and the avoidance of mTOR-dependent nutrient uptake promote the accumulation of α-KG/TET-dependent hydroxymethylation events within the *Foxp3* CNS2 cis-acting element of resting cells (step 1). (b) mTOR-dependent proliferation and chromosomal replication subsequently allow for the demethylation of all daughter-strand CNS2 CpG nucleotides as a consequence of the antagonism between DNMT1 and TET proteins, particularly when the locus is already partially demethylated (step 2). Taken together, this model now predicts that the degree of CpG demethylation acquired within the *Foxp3* CNS2 locus in step 1 specifies the anergic T-cell fate in step 2. Furthermore, the model offers the possibility that metabolic intervention can modulate the level of CNS2 methylation in anergic CD4 T cells and influence the differentiation of anergy-derived Foxp3 ^+^ Treg cells. In particular, future investigations of the metabolic regulation of the DNA methyltransferase DNMT1 and the TET methylcytosine dioxygenases in anergic CD4 T cells may yield key insights.

## References

[1] Mueller DL: Mechanisms maintaining peripheral tolerance. *Nat Immunol.* 2010;11(1):21–7. 10.1038/ni.1817 20016506

[2] Kitagawa Y Sakaguchi S: Molecular control of regulatory T cell development and function. *Curr Opin Immunol.* 2017;49:64–70. 10.1016/j.coi.2017.10.002 29065384

[3] Sakaguchi S Sakaguchi N Asano M: Immunologic self-tolerance maintained by activated T cells expressing IL-2 receptor alpha-chains (CD25). Breakdown of a single mechanism of self-tolerance causes various autoimmune diseases. *J Immunol.* 1995;155(3):1151–64. 7636184

[4] Chappert P Schwartz RH: Induction of T cell anergy: integration of environmental cues and infectious tolerance. *Curr Opin Immunol.* 2010;22(5):552–9. 10.1016/j.coi.2010.08.005 20869863 PMC2981408

[5] Kalekar LA Schmiel SE Nandiwada SL: CD4 ^+^ T cell anergy prevents autoimmunity and generates regulatory T cell precursors. *Nat Immunol.* 2016;17(3):304–14. 10.1038/ni.3331 26829766 PMC4755884

[6] Fontenot JD Gavin MA Rudensky AY: Foxp3 programs the development and function of CD4 ^+^CD25 ^+^ regulatory T cells. *Nat Immunol.* 2003;4(4):330–6. 10.1038/ni904 12612578

[7] Brunkow ME Jeffery EW Hjerrild KA: Disruption of a new forkhead/winged-helix protein, scurfin, results in the fatal lymphoproliferative disorder of the scurfy mouse. *Nat Genet.* 2001;27(1):68–73. 10.1038/83784 11138001

[8] Bennett CL Christie J Ramsdell F: The immune dysregulation, polyendocrinopathy, enteropathy, X-linked syndrome (IPEX) is caused by mutations of *FOXP3*. *Nat Genet.* 2001;27(1):20–1. 10.1038/83713 11137993

[9] Wildin RS Ramsdell F Peake J: X-linked neonatal diabetes mellitus, enteropathy and endocrinopathy syndrome is the human equivalent of mouse scurfy. *Nat Genet.* 2001;27(1):18–20. 10.1038/83707 11137992

[10] Zheng Y Josefowicz SZ Kas A: Genome-wide analysis of Foxp3 target genes in developing and mature regulatory T cells. *Nature.* 2007;445(7130):936–40. 10.1038/nature05563 17237761

[11] Arvey A van der Veeken J Samstein RM: Inflammation-induced repression of chromatin bound by the transcription factor Foxp3 in regulatory T cells. *Nat Immunol.* 2014;15(6):580–7. 10.1038/ni.2868 24728351 PMC4112080

[12] Gerriets VA Kishton RJ Johnson MO: Foxp3 and Toll-like receptor signaling balance T _reg_ cell anabolic metabolism for suppression. *Nat Immunol.* 2016;17(12):1459–66. 10.1038/ni.3577 27695003 PMC5215903

[13] Zeng H Yang K Cloer C: mTORC1 couples immune signals and metabolic programming to establish T _reg_-cell function. *Nature.* 2013;499(7459):485–90. 10.1038/nature12297 23812589 PMC3759242

[14] Floess S Freyer J Siewert C: Epigenetic control of the *foxp3* locus in regulatory T cells. *PLoS Biol.* 2007;5(2):e38. 10.1371/journal.pbio.0050038 17298177 PMC1783672

[15] Kim HP Leonard WJ: CREB/ATF-dependent T cell receptor-induced *FoxP3* gene expression: a role for DNA methylation. *J Exp Med.* 2007;204(7):1543–51. 10.1084/jem.20070109 17591856 PMC2118651

[16] Polansky JK Kretschmer K Freyer J: DNA methylation controls *Foxp3* gene expression. *Eur J Immunol.* 2008;38(6):1654–63. 10.1002/eji.200838105 18493985

[17] Zheng Y Josefowicz S Chaudhry A: Role of conserved non-coding DNA elements in the *Foxp3* gene in regulatory T-cell fate. *Nature.* 2010;463(7282):808–12. 10.1038/nature08750 20072126 PMC2884187

[18] Ohkura N Hamaguchi M Morikawa H: T cell receptor stimulation-induced epigenetic changes and Foxp3 expression are independent and complementary events required for Treg cell development. *Immunity.* 2012;37(5):785–99. 10.1016/j.immuni.2012.09.010 23123060

[19] Vahl JC Drees C Heger K: Continuous T cell receptor signals maintain a functional regulatory T cell pool. *Immunity.* 2014;41(5):722–36. 10.1016/j.immuni.2014.10.012 25464853

[20] Lal G Zhang N van der Touw W: Epigenetic regulation of Foxp3 expression in regulatory T cells by DNA methylation. *J Immunol.* 2009;182(1):259–73. 10.4049/jimmunol.182.1.259 19109157 PMC3731994

[21] Kim GD Ni J Kelesoglu N: Co-operation and communication between the human maintenance and *de novo* DNA (cytosine-5) methyltransferases. *EMBO J.* 2002;21(15):4183–95. 10.1093/emboj/cdf401 12145218 PMC126147

[22] Nair VS Song MH Ko M: DNA Demethylation of the Foxp3 Enhancer Is Maintained through Modulation of Ten-Eleven-Translocation and DNA Methyltransferases. *Mol Cells.* 2016;39(12):888–97. 10.14348/molcells.2016.0276 27989104 PMC5223106

[23] Yue X Trifari S Äijö T: Control of Foxp3 stability through modulation of TET activity. *J Exp Med.* 2016;213(3):377–97. 10.1084/jem.20151438 26903244 PMC4813667

[24] Josefowicz SZ Wilson CB Rudensky AY: Cutting edge: TCR stimulation is sufficient for induction of Foxp3 expression in the absence of DNA methyltransferase 1. *J Immunol.* 2009;182(11):6648–52. 10.4049/jimmunol.0803320 19454658

[25] Someya K Nakatsukasa H Ito M: Improvement of Foxp3 stability through CNS2 demethylation by TET enzyme induction and activation. *Int Immunol.* 2017;29(8):365–75. 10.1093/intimm/dxx049 29048538 PMC5890887

[26] Gerriets VA Kishton RJ Nichols AG: Metabolic programming and PDHK1 control CD4 ^+^ T cell subsets and inflammation. *J Clin Invest.* 2015;125(1):194–207. 10.1172/JCI76012 25437876 PMC4382238

[27] Michalek RD Gerriets VA Jacobs SR: Cutting edge: distinct glycolytic and lipid oxidative metabolic programs are essential for effector and regulatory CD4 ^+^ T cell subsets. *J Immunol.* 2011;186(6):3299–303. 10.4049/jimmunol.1003613 21317389 PMC3198034

[28] Delgoffe GM Kole TP Zheng Y: The mTOR kinase differentially regulates effector and regulatory T cell lineage commitment. *Immunity.* 2009;30(6):832–44. 10.1016/j.immuni.2009.04.014 19538929 PMC2768135

[29] Delgoffe GM Woo SR Turnis ME: Stability and function of regulatory T cells is maintained by a neuropilin-1-semaphorin-4a axis. *Nature.* 2013;501(7466):252–6. 10.1038/nature12428 23913274 PMC3867145

[30] Sun IH Oh MH Zhao L: mTOR Complex 1 Signaling Regulates the Generation and Function of Central and Effector Foxp3 ^+^ Regulatory T Cells. *J Immunol.* 2018;201(2):481–92. 10.4049/jimmunol.1701477 29884702 PMC6089237

[31] Morita M Gravel SP Chénard V: mTORC1 controls mitochondrial activity and biogenesis through 4E-BP-dependent translational regulation. *Cell Metab.* 2013;18(5):698–711. 10.1016/j.cmet.2013.10.001 24206664

[32] Wieman HL Wofford JA Rathmell JC: Cytokine stimulation promotes glucose uptake via phosphatidylinositol-3 kinase/Akt regulation of Glut1 activity and trafficking. *Mol Biol Cell.* 2007;18(4):1437–46. 10.1091/mbc.e06-07-0593 17301289 PMC1838986

[33] Ray JP Staron MM Shyer JA: The Interleukin-2-mTORc1 Kinase Axis Defines the Signaling, Differentiation, and Metabolism of T Helper 1 and Follicular B Helper T Cells. *Immunity.* 2015;43(4):690–702. 10.1016/j.immuni.2015.08.017 26410627 PMC4618086

[34] Wofford JA Wieman HL Jacobs SR: IL-7 promotes Glut1 trafficking and glucose uptake via STAT5-mediated activation of Akt to support T-cell survival. *Blood.* 2008;111(4):2101–11. 10.1182/blood-2007-06-096297 18042802 PMC2234050

[35] Frauwirth KA Riley JL Harris MH: The CD28 signaling pathway regulates glucose metabolism. *Immunity.* 2002;16(6):769–77. 10.1016/S1074-7613(02)00323-0 12121659

[36] Ducker GS Rabinowitz JD: One-Carbon Metabolism in Health and Disease. *Cell Metab.* 2017;25(1):27–42. 10.1016/j.cmet.2016.08.009 27641100 PMC5353360

[37] Ron-Harel N Santos D Ghergurovich JM: Mitochondrial Biogenesis and Proteome Remodeling Promote One-Carbon Metabolism for T Cell Activation. *Cell Metab.* 2016;24(1):104–17. 10.1016/j.cmet.2016.06.007 27411012 PMC5330619

[38] Chisolm DA Weinmann AS: Connections Between Metabolism and Epigenetics in Programming Cellular Differentiation. *Annu Rev Immunol.* 2018;36:221–46. 10.1146/annurev-immunol-042617-053127 29328786

[39] Colombo SL Palacios-Callender M Frakich N: Anaphase-promoting complex/cyclosome-Cdh1 coordinates glycolysis and glutaminolysis with transition to S phase in human T lymphocytes. *Proc Natl Acad Sci U S A.* 2010;107(44):18868–73. 10.1073/pnas.1012362107 20921411 PMC2973876

[40] MacIver NJ Michalek RD Rathmell JC: Metabolic regulation of T lymphocytes. *Annu Rev Immunol.* 2013;31:259–83. 10.1146/annurev-immunol-032712-095956 23298210 PMC3606674

[41] Jooss K Gjata B Danos O: Regulatory function of *in vivo* anergized CD4 ^+^ T cells. *Proc Natl Acad Sci U S A.* 2001;98(15):8738–43. 10.1073/pnas.151088898 11438696 PMC37505

[42] Buer J Lanoue A Franzke A: Interleukin 10 secretion and impaired effector function of major histocompatibility complex class II-restricted T cells anergized *in vivo*. *J Exp Med.* 1998;187(2):177–83. 10.1084/jem.187.2.177 9432975 PMC2212096

[43] Chen TC Cobbold SP Fairchild PJ: Generation of anergic and regulatory T cells following prolonged exposure to a harmless antigen. *J Immunol.* 2004;172(10):5900–7. 10.4049/jimmunol.172.10.5900 15128770

[44] Pletinckx K Vaeth M Schneider T: Immature dendritic cells convert anergic nonregulatory T cells into Foxp3 ^-^IL-10 ^+^ regulatory T cells by engaging CD28 and CTLA-4. *Eur J Immunol.* 2015;45(2):480–91. 10.1002/eji.201444991 25382658

[45] Kalekar LA Mueller DL: Relationship between CD4 Regulatory T Cells and Anergy *In Vivo*. *J Immunol.* 2017;198(7):2527–33. 10.4049/jimmunol.1602031 28320913 PMC5363282

[46] Bailey-Bucktrout SL Martinez-Llordella M Zhou X: Self-antigen-driven activation induces instability of regulatory T cells during an inflammatory autoimmune response. *Immunity.* 2013;39(5):949–62. 10.1016/j.immuni.2013.10.016 24238343 PMC3912996

[47] Komatsu N Mariotti-Ferrandiz ME Wang Y: Heterogeneity of natural Foxp3 ^+^ T cells: a committed regulatory T-cell lineage and an uncommitted minor population retaining plasticity. *Proc Natl Acad Sci U S A.* 2009;106(6):1903–8. 10.1073/pnas.0811556106 19174509 PMC2644136

[48] Feng Y Arvey A Chinen T: Control of the inheritance of regulatory T cell identity by a *cis* element in the *Foxp3* locus. *Cell.* 2014;158(4):749–63. 10.1016/j.cell.2014.07.031 25126783 PMC4151558

[49] Kim HJ Barnitz RA Kreslavsky T: Stable inhibitory activity of regulatory T cells requires the transcription factor Helios. *Science.* 2015;350(6258):334–9. 10.1126/science.aad0616 26472910 PMC4627635

[50] Miyao T Floess S Setoguchi R: Plasticity of Foxp3 ^+^ T cells reflects promiscuous Foxp3 expression in conventional T cells but not reprogramming of regulatory T cells. *Immunity.* 2012;36(2):262–75. 10.1016/j.immuni.2011.12.012 22326580

[51] Cantó C Auwerx J: Targeting sirtuin 1 to improve metabolism: all you need is NAD ^+^? *Pharmacol Rev.* 2012;64(1):166–87. 10.1124/pr.110.003905 22106091 PMC3616312

[52] van Loosdregt J Vercoulen Y Guichelaar T: Regulation of Treg functionality by acetylation-mediated Foxp3 protein stabilization. *Blood.* 2010;115(5):965–74. 10.1182/blood-2009-02-207118 19996091

[53] Beier UH Wang L Bhatti TR: Sirtuin-1 targeting promotes Foxp3 ^+^ T-regulatory cell function and prolongs allograft survival. *Mol Cell Biol.* 2011;31(5):1022–9. 10.1128/MCB.01206-10 21199917 PMC3067815

[54] Zheng Y Delgoffe GM Meyer CF: Anergic T cells are metabolically anergic. *J Immunol.* 2009;183(10):6095–101. 10.4049/jimmunol.0803510 19841171 PMC2884282

[55] Ananieva EA Patel CH Drake CH: Cytosolic branched chain aminotransferase (BCATc) regulates mTORC1 signaling and glycolytic metabolism in CD4 ^+^ T cells. *J Biol Chem.* 2014;289(27):18793–804. 10.1074/jbc.M114.554113 24847056 PMC4081922

[56] Gu X Orozco JM Saxton RA: SAMTOR is an S-adenosylmethionine sensor for the mTORC1 pathway. *Science.* 2017;358(6364):813–8. 10.1126/science.aao3265 29123071 PMC5747364

[57] van Loosdregt J Brunen D Fleskens V: Rapid temporal control of Foxp3 protein degradation by sirtuin-1. *PLoS One.* 2011;6(4):e19047. 10.1371/journal.pone.0019047 21533107 PMC3080399

[58] Zhang J Lee SM Shannon S: The type III histone deacetylase Sirt1 is essential for maintenance of T cell tolerance in mice. *J Clin Invest.* 2009;119(10):3048–58. 10.1172/JCI38902 19729833 PMC2752073

